# Reading, singing, and storytelling: the impact of caregiver-child interaction and child access to books and preschool on early childhood development in Ghana

**DOI:** 10.1038/s41598-023-38439-5

**Published:** 2023-08-23

**Authors:** Shamsudeen Mohammed, Agani Afaya, Alhassan Sibdow Abukari

**Affiliations:** 1 Department of Non-Communicable Disease Epidemiology, Faculty of Epidemiology and Population Health, London School of Hygiene and Tropical Medicine, London, UK; 2 Mo-Im Kim Nursing Research Institute, College of Nursing, Yonsei University, 50-1, Yonsei-Ro, Seodaemun-Gu, Seoul, 03722 South Korea; 3 Department of Nursing, School of Nursing and Midwifery, University of Health and Allied Sciences, Ho, Ghana; 4 Department of General Nursing, School of Nursing and Midwifery, Wisconsin International University College, North Legon, Postal Address: Box LG 751, Accra, Ghana

**Keywords:** Health care, Medical research, Risk factors

## Abstract

Optimal early childhood development (ECD) is crucial in shaping future academic and economic accomplishments. Recognising its profound influence, the United Nations has included a specific target and indicator related to ECD in the Sustainable Development Goals to ensure universal access to high-quality ECD for all preschoolers by 2030. This study investigated whether caregiver-child engagements, early stimulation, and learning opportunities are associated with ECD among children aged 36–59 months. Data on 6752 children were pooled from the 2011 and 2017 Ghana Multiple Indicator Cluster Surveys. The ECD outcomes of interest were literacy-numeracy, social-emotional, learning-cognitive, and physical development, measured with UNICEF's Early Childhood Development Index (ECDI). Binary logistic regression assessed the effects of various caregiver-child engagements and early learning opportunities on the ECD outcomes. We found that most children were developmentally on track in the physical (95.0%) and learning-cognitive (86.4%) domains, but fewer were on track in the literacy-numeracy (36.7%) and social-emotional (68.6%) domains. Reading to or with a child (aOR 1.72; 95% CI 1.35–2.19), telling them stories (aOR 1.61; 95% CI 1.26–2.04), counting or drawing with them (aOR 1.63; 95% CI 1.30–2.04) and a child’s attendance at preschool (aOR 4.62; 95% CI 3.34–6.38) were associated with a higher odds for on track literacy-numeracy development. Playing with a child was associated with higher odds of on-track social-emotional development (aOR 1.29; 95% CI 1.04–1.59), physical development (aOR 1.61; 95% CI 1.01–2.55), and learning-cognitive development (aOR 1.51; 95% CI 1.14–2.00). However, singing songs to or with a child (aOR 0.78; 95% CI 0.62–0.89) and taking a child outside the home (aOR 0.78; 95% CI 0.64–0.95) were associated with lower odds for on track social-emotional development. Access to children's books at home was associated with higher odds for on track literacy-numeracy and social-emotional development. In subgroup analysis, some observed associations varied depending on a child's residence (urban or rural). Our findings confirmed that caregiver-child  interaction and early learning opportunities could increase a child’s likelihood of achieving early developmental milestones.

## Introduction

In low- and middle-income countries, a staggering 250 million children under age five are at risk of suboptimal development, with most of these at-risk children living in South Asia and sub-Saharan Africa^1–3^. However, research has shown that children can escape developmental delays with appropriate early stimulation and learning opportunities, including responsive caregiving and optimal nutrition^4^. In the first few years after birth, the maturing human brain has the remarkable ability to absorb and integrate experiences into its developing structure and functioning, affecting the formation of neural connections and the development of cognitive, language, emotional, and social skills^5^. As such, during this critical period of neuroplasticity, children are particularly sensitive to the influence of their environment and interactions with people around them^3,5^. For example, research has shown that when a child is neglected, abused, or exposed to violence at a young age, their risk of developing physical, social, and mental health issues later in life increases^6,7^. In contrast, stimulating and supportive environments, such as those that provide opportunities for learning and social interaction, can promote positive brain development and enhance language, cognitive, and social-emotional abilities^8,9^.

Previous studies have demonstrated that activities like reading, storytelling, counting, drawing and singing can improve children's literacy and numeracy skills and language and cognitive development, albeit with some inconsistencies^10–17^. Using data from the Young Lives Longitudinal Survey in Ethiopia, Woldehanna showed that preschool attendance was associated with 31% higher scores on vocabulary tests and 23% on a quantitative test compared to not attending preschool^18^. In a Brazilian study of over 6000 children under age five, children whose caregivers read books to them and engaged them in stimulating activities were more likely to reach their developmental milestones than those who did not experience these early stimulations^19^. In addition, the study found that ownership of books at home was associated with higher attainment of developmental milestones.

Despite the importance of early stimulation and learning opportunities for early childhood development, few studies have investigated these associations in Ghana. Most studies investigating the risk and protective factors for early childhood development in Ghana often focus on the effects of early nutrition, childhood diseases, and poverty^25–28^. Although previous studies from other low- and middle-income contexts have offered valuable insights into early childhood development^29^, they fail to encompass the distinctive challenges and opportunities Ghanaian children face. According to UNICEF, many Ghanaian children lack fundamental numeracy and literacy skills^21^. In response to this concern, the Ghanaian government has initiated various programs to enhance educational accessibility and support caregivers. One such program is the Livelihood Empowerment Against Poverty (LEAP) program, which was introduced in 2008^22^. One of the primary objectives of the LEAP program was to remove financial barriers to education. It resulted in a 10% reduction in primary school absenteeism and a 7% increase in secondary school enrolment^23^. Furthermore, the introduction of the free senior high school program in 2017 expanded opportunities for secondary education. In addition, preschool and primary education are free in all public schools in Ghana. Notwithstanding these policy efforts, there are still significant challenges in the early childhood development sector in Ghana. Access to high-quality preschool education remains a major concern, particularly in rural areas where many children are deprived of early childhood education opportunities^24^.

Early childhood development is heavily influenced by culture and context, and Ghana has unique parenting practices and childrearing norms that may impact development differently than in other countries^30^. One thing that sets Ghana apart from many other societies is the central role that extended family members play in childrearing. Raising a child is often shared among extended family members such as grandparents, aunts, and uncles^30–32^. While this is true in all Ghanaian communities, in more rural and traditional settings, the responsibility of childrearing extends to the entire community. Neighbours, friends, and relatives all help to guide, discipline, and nurture children. Respect for authority figures, particularly parents and elders, is another distinguishing feature of Ghanaian parenting^30^. This emphasis on respect influences the behaviour and social interactions of children. For instance, it can help instil obedience and discipline in children, but it also has the potential to stifle their independence and self-expression.

In light of Ghana's unique parenting practices, certain factors considered important for early childhood development on a global scale may not hold the same relevance within the Ghanaian context. The analysis of Ghana-specific data yields findings representative of the Ghanaian population and can be used to inform culturally relevant policies. Moreover, evidence-based policymaking requires data specific to the context in which policies are being developed, so Ghanaian policymakers are more likely to prioritise and act on research specific to their context. Therefore, we investigated the impact of various forms of caregiver-child engagement, early stimulation, and learning opportunities on early childhood development among children aged 36–59 months in Ghana.

## Methods

The data for this study came from two nationally representative cross-sectional household surveys—the 2011 and 2017 Ghana Multiple Indicator Cluster Surveys (MICS). The Ghana Statistical Service implemented the surveys in collaboration with government agencies and ministries. To ensure representativeness at the national and sub-national levels, stratified multistage sampling was used to select households and participants for the surveys. First, the administrative regions of Ghana were stratified into rural and urban areas, and a probability proportional to size method was used to select census enumeration areas in each stratum. The enumeration areas were used as the primary sampling units. Systematic random sampling was used to select a specified number of households (15 households in the 2011 survey and 20 in the 2017 survey) from each primary sampling unit for interviews. Several questionnaires were administered in each household, including a household questionnaire to collect data on the basic characteristics of the household and household members, an individual women's questionnaire to understand the situation of women aged 15–49, and an under-5 questionnaire to assess the health, nutrition, growth and development of all children under-5 living in the household. The under-five questionnaire was administered to the mothers or primary caretakers of the children. Also, a men's questionnaire was administered in every second household (every third household in the 2011 survey) to men aged 15–49. In addition to the English version of the questionnaires, they were also translated into the four main Ghanaian languages (Akan, Dagbanli, Ewe, and Ga). The fieldwork for the 2011 survey was conducted between September 2011 and December 2011, whiles the 2017 survey began in October 2017 and ended in January 2018. The response rates for each of the surveys were over 90%. The present study analysed data on children aged 36–59 months with complete information on early childhood development indicators.

### Outcome

Early Childhood Development (ECD) was the outcome of this study. It was measured using the Early Childhood Development Index (ECDI) developed by the United Nations International Children's Emergency Fund (UNICEF). The ECDI includes ten questions to capture children's achievement in four critical developmental domains: literacy-numeracy skills (3 items), physical growth (2 items), learning-cognition (2 items), and social-emotional development (3 items). The ten questions of the ECDI and the scoring approach implemented in this study are outlined in Table 1. Mothers or primary caregivers of children aged 36–59 months were the primary respondents, and their responses were recorded in binary, 'Yes' or 'No'. In compliance with UNICEF's guidelines^33^, each item of the ECDI scale was scored 1 if true for the child and 0 if otherwise. Achievements in the four domains were appraised separately to determine if a child was developmentally on track. A child was considered developmentally on track in the literacy-numeracy and socio-emotional domains if at least two of the domains items were true. Children were developmentally on track in the physical growth and learning-cognition domains if one or both of the domains items were true.

**Table 1:** Early childhood development index.

Literacy-numeracy	
1	Can (name) identify or name at least ten letters of the alphabet?
2	Can (name) read at least four simple, popular words?
3	Does (name) know the name and recognise the symbol of all numbers from 1 to 10?
Physical	
4	Can (name) pick up a small object with two fingers, like a stick or a rock from the ground?
5	Is (name) sometimes too sick to play? (reverse coded)
Approaches to learning	
6	Does (name) follow simple directions on how to do something correctly?
7	When given something to do, is (name) able to do it independently?
Social-emotional	
8	Does (name) get along well with other children?
9	Does (name) kick, bite or hit other children or adults? (reverse coded)
10	Does (name) get distracted easily? (reverse coded)

### Exposures

Mothers or primary caregivers were asked if the mother, father, or other adult household members aged 15 or older had engaged in the following early stimulating activities with children under the age of five in the three days before the interview: (1) Read books or looked at picture books with the child, (2) Told stories to the child, (3) Sang songs to or with the child, including lullabies (4) Took the child outside the home, (5) Played with the child, and (6) Named, counted, or drew things for or with the child. Each question was coded 1 if a child experienced the activity and 0 if otherwise. In addition, mothers or primary caregivers were asked about the number of children's books or picture books they have for children under five (*"How many children's books or picture books do you have for (name)?").* Responses to the question were recoded as 'None', '1–2 books' and 'three or more books' for the present analysis. To assess preschool attendance, mothers or primary caregivers of children aged 3 and 4 were asked if the child ever attended any early childhood education programme, such as nursery, preschool or kindergarten (KG)—yes or no responses were recorded.

### Data analysis

Descriptive statistics were used to summarise the characteristics of the study sample. We estimated the weighted percentage of children aged 36–59 months developmentally on track in the literacy-numeracy, social-emotional, physical growth, and learning-cognitive development domains. Binary logistic regression was used to estimate odds ratios with 95% confidence intervals for the association of various caregiver-child engagements (reading, singing, storytelling, and playing), access to books, and preschool attendance with early childhood development. To account for potential confounding factors, adjusted odds ratios were calculated using multivariable binary logistic regression in a complete case analysis. In the adjusted analysis, we controlled for the child's age, sex, birth order, place of residence, household wealth, maternal age, marital status, education, multiple births, previous birth interval, ethnicity, and paternal education. However, due to missing data on "maternal report of child size at birth" for 63.7% of the participants, it was not included in the multivariable models. To adjust for the oversampling in certain enumeration areas and to estimate appropriate standard errors, we accounted for the clustering and stratification in the design of the surveys and applied individual survey weights using the complex survey functions of Stata 17. We considered a p-value of less than 0.05 to indicate statistically significant.

## Results

Overall, 6752 children aged 36–59 months were included in the analysis. Table 2 presents the characteristics of the study sample. Girls constituted 50.5% of the total sample, and just under half (48.9%) of the children were large at birth. Nearly all the children were singletons (96.0%), and 19.3% were firstborns. At the time of the interview, most mothers were between 20 and 34 years old, married, and lived in rural areas of Ghana, and only 34.9% lived in high-income households. Although 54.6% of mothers had primary education, one-third never attended school, and only 4.0% had higher education. On the other hand, 20.9% of fathers had higher education, and 26.6% never attended school.

**Table 2:** Distribution of participants characteristics.

	Number (weighted)	Percent (weighted)
Child’s sex		
Male	3337	49.5
Female	3410	50.5
Maternal report of child size at birth		
Large	1191	48.9
Average	918	37.7
Small	329	13.5
Multiple births		
Yes	223	4.0
No	5359	96.0
Birth order		
1	1076	19.3
2–3	2056	36.8
4 +	2450	43.9
Previous birth interval		
< 2 years	1750	31.3
2–3 years	2395	42.9
4 + years	1437	25.8
Household income		
Low	3009	44.6
Middle	1384	20.5
High	2354	34.9
Place of residence		
Urban	2904	43.0
Rural	3844	57.0
Maternal age		
< 20	48	0.8
20–34	3532	59.2
35–49	2383	40.0
Mothers marital status		
Unmarried	2024	33.9
Married	3938	66.1
Maternal education		
Never attended school	2132	33.3
Primary	3502	54.6
Secondary	515	8.0
Higher	259	4.0
Paternal education		
Never attended school	1407	26.6
Primary	2242	42.5
Secondary	526	10.0
Higher	1105	20.9

Unweighted missing observations (multiple births [n = 1199, 17.8%], birth order [n = 1199, 17.8%], previous birth interval [n = 1199, 17.8%], maternal report of child size at birth = [n = 4298, 63.7%], maternal age [n = 846, 12.5%], mothers marital status [n = 846, 12.5%], maternal education [n = 375, 5.6]).

Note: “previous birth interval” include “first births” in the “ < 2 years” category.

### Association of caregiver-child engagements, early stimulation, and learning opportunities with early childhood development among children aged 36–59 months

**Figure 1:**
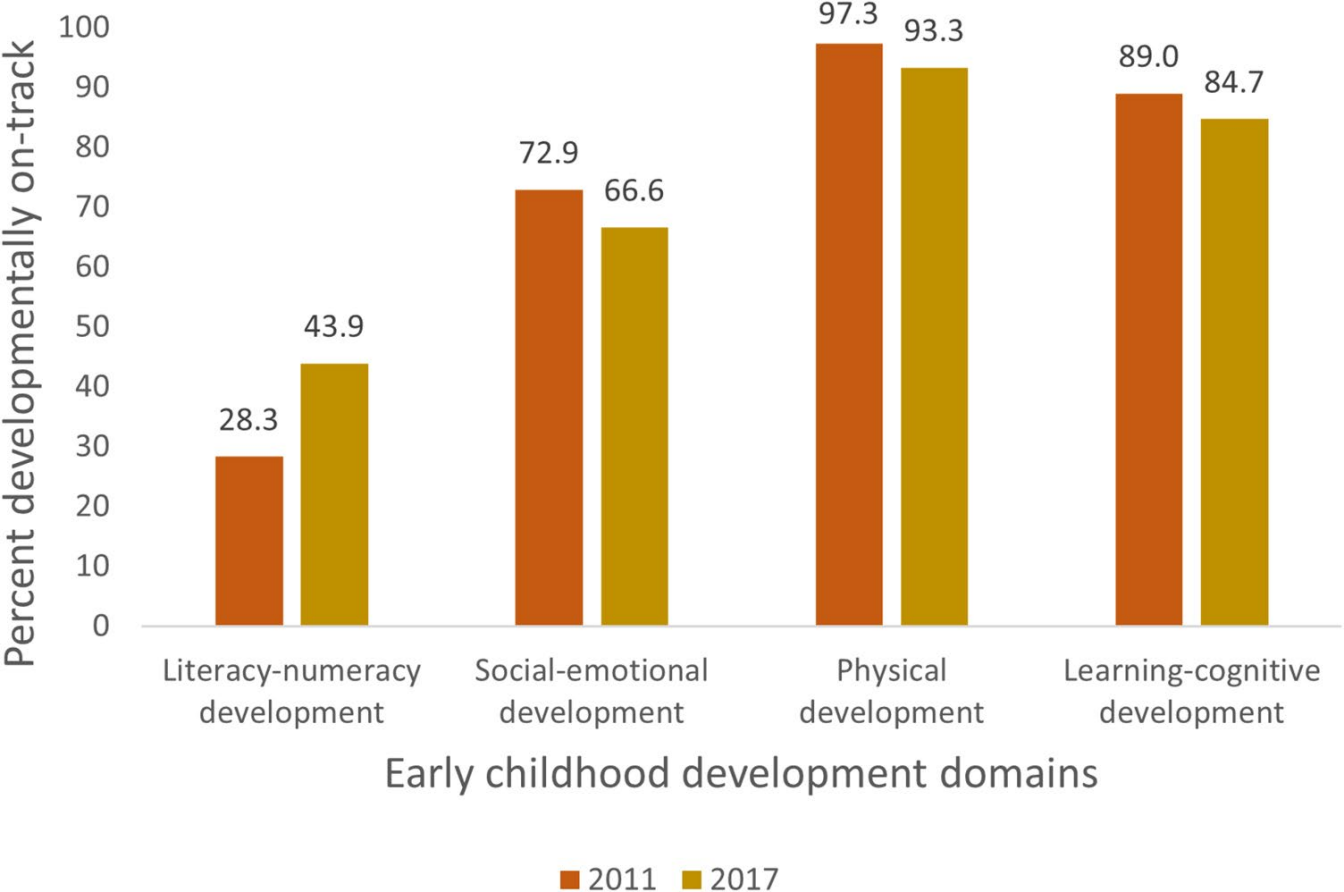
Trends in the percent of children developmentally on track in literacy-numeracy, social-emotional, learning-cognition, and physical development domains between 2011 and 2017.

Overall, 36.7% of the children were developmentally on track in literacy-numeracy, 68.6% in social-emotional development, 95.0% in physical development, and 86.4% in learning-cognitive development (Table 3). Figure 1 shows that the percentage of children developmentally on track in the literacy-numeracy domain rose from 28.3% in 2011 to 43.9% in 2017. However, during the same period, there was a decline in the percentage of children who were developmentally on track in the social-emotional (72.9% in 2011 to 66.6% in 2017), physical (97.3% in 2011 to 93.3% in 2017), and learning-cognitive (89.0% in 2011 to 84.7% in 2017) domains.

**Table 3:** Distribution of caregiver-child reading, singing, storytelling and playing engagements and access to books and preschool attendance across early childhood development indicators.

	On track in literacy-numeracy development		On track in social-emotional development		On track in physical development		On track in learning/cognition development	
	No (%)	Yes (%)	No (%)	Yes (%)	No (%)	Yes (%)	No (%)	Yes (%)
All	4273 (63.3)	2474 (36.7)	2121 (31.4)	4626 (68.6)	341 (5.0)	6407 (95.0)	920 (13.6)	5828 (86.4)
Read books or looked at picture books with child								
Yes	946 (43.5)	1228 (56.5)	646 (29.7)	1528 (70.3)	108 (5.0)	2066 (95.0)	270 (12.4)	1904 (87.6)
No	3327 (72.8)	1246 (27.2)	1475 (32.3)	3098 (67.7)	233 (5.1)	4341 (94.9)	649 (14.2)	3924 (85.8)
Told stories to child								
Yes	1007 (49.1)	1043 (50.9)	656 (32.0)	1393 (68.0)	89 (4.3)	1961 (95.7)	281 (13.7)	1769 (86.3)
No	3266 (69.5)	1432 (30.5)	1465 (31.2)	3233 (68.8)	252 (5.4)	4445 (94.6)	639 (13.6)	4059 (86.4)
Named, counted, or drew things for or with child								
Yes	1248 (46.9)	1411 (53.1)	844 (31.7)	1815 (68.3)	130 (4.9)	2529 (95.1)	302 (11.4)	2357 (88.6)
No	3025 (74.0)	1063 (26.0)	1277 (31.2)	2811 (68.8)	211 (5.2)	3877 (94.8)	618 (15.1)	3470 (84.9)
Sang songs to or with child								
Yes	1953 (56.6)	1494 (43.4)	1153 (33.4)	2294 (66.6)	167 (4.8)	3281 (95.2)	480 (13.9)	2967 (86.1)
No	2320 (70.3)	980 (29.7)	968 (29.3)	2332 (70.7)	174 (5.3)	3126 (94.7)	439 (13.3)	2861 (96.7)
Took child outside the home								
Yes	2452 (62.0)	1505 (38.0)	1318 (33.3)	2639 (66.7)	177 (4.5)	3780 (95.5)	536 (13.5)	3421 (86.5)
No	1821 (65.3)	969 (34.7)	803 (28.8)	1987 (71.2)	164 (5.9)	2627 (94.1)	384 (13.8)	2406 (86.2)
Played with child								
Yes	3183 (61.2)	2020 (38.8)	1596 (30.7)	3607 (69.3)	238 (4.6)	4965 (95.4)	656 (12.6)	4547 (87.4)
No	1090 (70.6)	454 (29.4)	525 (34.0)	1020 (66.0)	103 (6.7)	1441 (93.3)	263 (17.1)	1281 (82.9)
Ever attended preschool								
Yes	2469 (51.9)	2287 (48.1)	1435 (30.2)	3321 (69.8)	247 (5.2)	4509 (94.8)	558 (11.7)	4198 (88.3)
No	1797 (90.5)	188 (9.5)	681 (34.3)	1303 (65.7)	89 (4.5)	1895 (95.5)	354 (17.9)	1630 (82.1)
Number of books at home								
None	3707 (73.1)	1362 (26.9)	1684 (33.2)	3385 (66.8)	268 (5.3)	4800 (94.7)	758 (15.0)	4311 (85.0)
1–2 books	351 (41.0)	506 (41.0)	237 (27.6)	621 (72.4)	44 (5.1)	814 (94.9)	94 (11.0)	764 (89.0)
3 or more books	214 (26.1)	606 (26.1)	201 (24.5)	620 (75.5)	28 (3.5)	792 (96.5)	68 (8.3)	753 (91.7)

### Literacy-numeracy development

**Figure 2:**
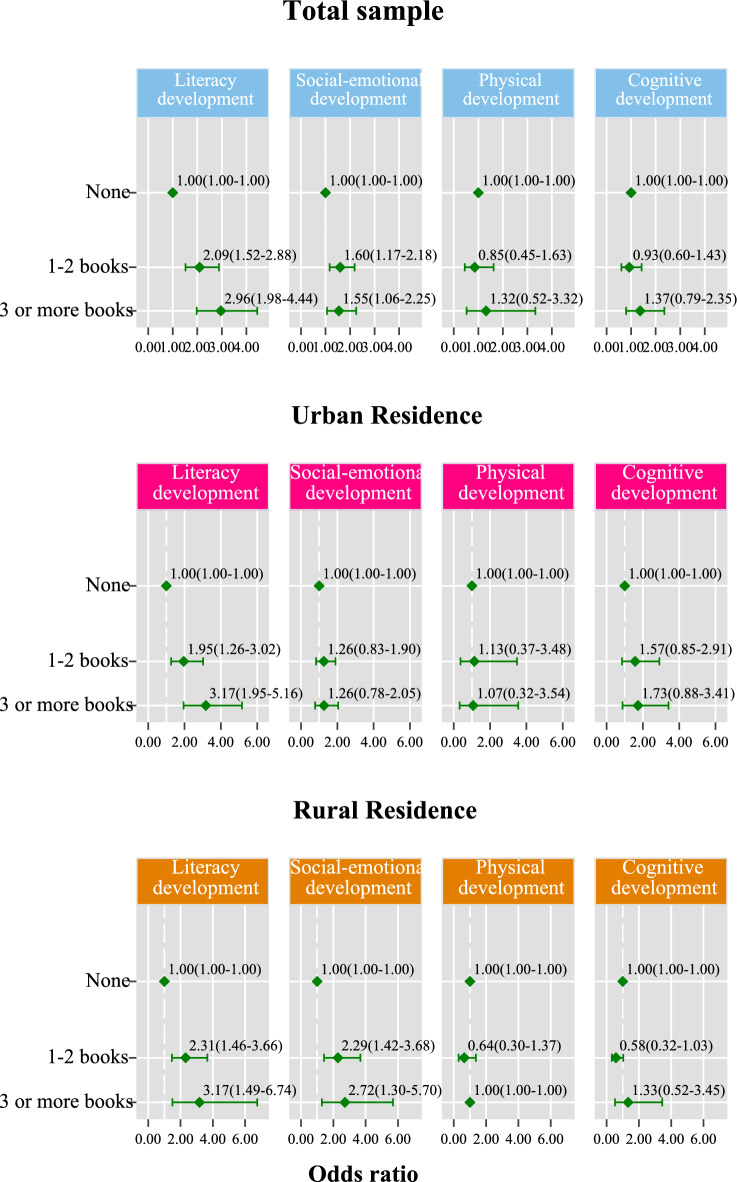
Adjusted association between access to books at home and early childhood development.

In adjusted analysis, reading to or with a child (aOR 1.72; 95% CI 1.35–2.19), telling them stories (aOR 1.61; 95% CI 1.26–2.04), and counting or drawing with them (aOR 1.63; 95% CI 1.30–2.04) were associated with a higher odds of on track literacy-numeracy development than not engaging them in these activities (Table 4). In addition, children who attended preschool had 4.62 (95% CI 3.34–6.38) higher odds of being on track in the literacy-numeracy domain than those who never attended preschool. The number of children's books at home had a dose–response effect on literacy-numeracy development. Children who lived in households with 1–2 and three or more children's books had 2.09 (95% CI 1.52–2.88) and 2.96 (95% CI 1.98–4.44) higher odds of being developmentally on track in the literacy-numeracy domain, respectively, than those who did not have children's books at home (Fig. 2).

**Table 4:** The association of caregiver-child reading, singing, storytelling and playing engagements and access to books and preschool attendance with early childhood development (n = 4550).

	On track literacy-numeracy development	On track social-emotional development	On track physical development	On track learning/cognition development
Read books or looked at picture books with child				
Unadjusted odds ratio (95% CI)	3.47 (2.93–4.11)	1.13 (0.95–1.33)	1.03 (0.74–1.42)	1.17 (0.92–1.47)
Adjusted odds ratio (95% CI)	1.72 (1.35–2.19)	1.12 (0.90–1.39)	0.92 (0.55–1.54)	0.91 (0.65–1.26)
Told stories to child				
Unadjusted odds ratio (95% CI)	2.36 (1.99–2.80)	0.96 (0.81–1.14)	1.25 (0.89–1.77)	0.99 (0.78–1.25)
Adjusted odds ratio (95% CI)	1.61 (1.26–2.04)	0.95 (0.77–1.18)	1.02 (0.67–1.55)	0.85 (0.64–1.14)
Named, counted, or drew things for or with child				
Unadjusted odds ratio (95% CI)	3.22 (2.71–3.82)	0.98 (0.84–1.14)	1.06 (0.77–1.47)	1.39 (1.11–1.74)
Adjusted odds ratio (95% CI)	1.63 (1.30–2.04)	0.96 (0.78–1.16)	0.99 (0.59–1.68)	1.20 (0.88–1.64)
Sang songs to or with child				
Unadjusted odds ratio (95% CI)	1.81 (1.56–2.11)	0.83 (0.71–0.96)	1.09 (0.82–1.46)	0.95 (0.77–1.17)
Adjusted odds ratio (95% CI)	1.09 (0.89–1.34)	0.78 (0.62–0.89)	1.07 (0.73–1.59)	0.87 (0.68–1.12)
Took child outside the home				
Unadjusted odds ratio (95% CI)	1.15 (0.95–1.40)	0.81 (0.68–0.96)	1.33 (0.99–1.79)	1.02 (0.82–1.26)
Adjusted odds ratio (95% CI)	0.95 (0.72–1.27)	0.78 (0.64–0.95)	1.24 (0.83–1.85)	0.90 (0.69–1.17)
Played with child				
Unadjusted odds ratio (95% CI)	1.52 (1.25–1.85)	1.16 (0.97–1.40)	1.49 (1.05–2.11)	1.42 (1.14–1.78)
Adjusted odds ratio (95% CI)	1.08 (0.81–1.44)	1.29 (1.04–1.59)	1.61 (1.01–2.55)	1.51 (1.14–2.00)
Ever attended preschool				
Unadjusted odds ratio (95% CI)	8.87 (6.83–11.53)	1.21 (1.03–1.42)	0.86 (0.62–1.18)	1.64 (1.34–2.00)
Adjusted odds ratio (95% CI)	4.62 (3.34–6.38)	1.19 (0.96–1.46)	0.68 (0.41–1.13)	1.26 (0.97–1.65)

Reference group for all variables = No.

In the subgroup analysis by residence type, the association between reading to or with a child, telling them stories, and counting or drawing with them and on track literacy-numeracy development among children in rural areas was similar to the substantive results (Table 5). However, among urban residents, only reading was associated with on track literacy-numeracy development, while telling stories and counting or drawing were not. The positive association between preschool attendance and on track literacy-numeracy development was stronger among children in rural areas (aOR 6.26; 95% CI 4.22–9.29) than children in urban areas (aOR 3.18; 95% CI 1.93–5.22). Access to children’s books at home was associated with literacy-numeracy development in both urban and rural areas (Fig. 2).

**Table 5:** The association of caregiver-child reading, singing, storytelling and playing engagements and access to books and preschool attendance with early childhood development by urban (n = 1457) and urban (n = 3379) residence.

	On track literacy-numeracy development		On track social-emotional development		On track physical development		On track learning/cognition development	
	Urban	Rural	Urban	Rural	Urban	Rural	Urban	Rural
Read books or looked at picture books with child								
Unadjusted odds ratio (95% CI)	2.49 (1.92–3.22)	3.61 (2.88–4.53)	1.14 (0.88–1.46)	1.07 (0.85–1.36)	1.28 (0.77–2.12)	0.80 (0.52–1.24)	1.35 (0.98–1.85)	0.89 (0.65–1.24)
Adjusted odds ratio (95% CI)	1.52 (1.09–2.11)	1.95 (1.39–2.74)	1.12 (0.80–1.57)	1.18 (0.88–1.57)	1.80 (0.74–4.43)	0.62 (0.35–1.10)	1.29 (0.82–2.04)	0.71 (0.46–1.10)
Told stories to child								
Unadjusted odds ratio (95% CI)	2.03 (1.55–2.66)	2.46 (1.96–3.08)	0.93 (0.71–1.21)	0.97 (0.78–1.20)	1.10 (0.64–1.90)	1.36 (0.87–2.14)	1.32 (0.86–2.02)	0.78 (0.59–1.03)
Adjusted odds ratio (95% CI)	1.27 (0.88–1.84)	1.94 (1.44–2.62)	0.86 (0.62–1.19)	1.03 (0.78–1.36)	0.72 (0.36–1.42)	1.26 (0.72–2.20)	1.11 (0.66–1.88)	0.73 (0.52–1.02)
Named, counted, or drew things for or with child								
Unadjusted odds ratio (95% CI)	2.33 (1.78–3.07)	3.25 (2.62–4.02)	0.92 (0.72–1.16)	0.98 (0.81–1.20)	1.27 (0.79–2.03)	0.87 (0.55–1.38)	1.49 (1.05–2.11)	1.15 (0.85–1.57)
Adjusted odds ratio (95% CI)	1.27 (0.92–1.75)	2.05 (1.52–2.78)	0.82 (0.61–1.11)	1.09 (0.83–1.42)	1.47 (0.63–3.45)	0.79 (0.42–1.50)	1.17 (0.74–1.88)	1.21 (0.80–1.84)
Sang songs to or with child								
Unadjusted odds ratio (95% CI)	1.49 (1.19–1.87)	1.89 (1.51–2.37)	0.74 (0.57–0.94)	0.88 (0.73–1.06)	1.09 (0.82–1.46)	1.09 (0.82–1.46)	0.83 (0.57–1.21)	0.95 (0.74–1.21)
Adjusted odds ratio (95% CI)	0.94 (0.69–1.29)	1.22 (0.91–1.63)	0.64 (0.45–0.91)	0.82 (0.66–1.02)	1.50 (0.74–3.07)	0.89 (0.55–1.42)	0.72 (0.45–1.15)	0.92 (0.68–1.23)
Took child outside the home								
Unadjusted odds ratio (95% CI)	1.00 (0.74–1.36)	1.28 (1.04–1.58)	0.78 (0.58–1.04)	0.83 (0.67–1.02)	1.14 (0.68–1.92)	1.46 (1.03–2.08)	1.07 (0.71–1.62)	1.06 (0.79–1.42)
Adjusted odds ratio (95% CI)	0.81 (0.52–1.25)	1.14 (0.84–1.54)	0.74 (0.52–1.05)	0.79 (0.62–1.00)	1.49 (0.75–2.98)	1.14 (0.70–1.86)	0.74 (0.46–1.21)	0.98 (0.72–1.33)
Played with child								
Unadjusted odds ratio (95% CI)	1.36 (1.02–1.81)	1.40 (1.08–1.83)	1.15 (0.77–1.72)	1.29 (0.99–1.66)	2.07 (1.23–3.49)	1.20 (0.76–1.91)	1.09 (0.72–1.67)	1.50 (1.15–1.96)
Adjusted odds ratio (95% CI)	1.13 (0.73–1.73)	1.01 (0.72–1.42)	1.13 (0.75–1.71)	1.30 (1.02–1.67	3.37 (1.68–6.74)	1.06 (0.62–1.84)	1.04 (0.56–1.93)	1.69 (1.24–2.31)
Attended preschool								
Unadjusted odds ratio (95% CI)	5.68 (3.72–8.68)	9.96 (7.36–13.49)	1.41 (1.03–1.95)	1.09 (0.91–1.32)	1.32 (0.75–2.33)	0.66 (0.44–0.98)	1.58 (1.04–2.40)	1.48 (1.17–1.89)
Adjusted odds ratio (95% CI)	3.18 (1.93–5.22)	6.26 (4.22–9.29)	1.44 (0.90–2.29)	1.11 (0.89–1.39)	1.10 (0.33–3.66)	0.55 (0.33–0.92)	1.23 (0.71–2.15)	1.23 (0.91–1.68)

Reference group for all variables = No.

### Social-emotional development

In the adjusted analysis, singing songs to or with a child (aOR 0.78; 95% CI 0.62–0.89) and taking a child outside the home (aOR 0.78; 95% CI 0.64–0.95) were associated with a reduced likelihood of on track social-emotional development (Table 4). However, playing with the child was associated with a higher likelihood of on track social-emotional development than not playing with the child (aOR 1.29; 95% CI 1.04–1.59). Children with access to 1–2 books (aOR 1.60; 95% CI 1.17–2.18) and those with access to three or more books (aOR 1.55; 95% CI 1.06–2.25) at home were more likely to be developmentally on track in the social-emotional domain than those with no access to books at home (Fig. 2).

In the subgroup analysis by residence type, only singing songs to or with a child was associated with a reduced likelihood of on track social-emotional development (aOR 0.64; 95% CI 0.45–0.91) among children in urban areas (Table 5). There was no evidence of an association between taking a child outside the home, playing with the child, and on track social-emotional development among children in urban areas. However, among those in rural areas, there was some evidence that singing songs to or with a child (aOR 0.82; 95% CI 0.66–1.02) and taking a child outside the home (aOR 0.79; 95% CI 0.62–1.00) were associated with a reduced likelihood of on track development in the social-emotional domain while playing with a child (aOR 1.30; 95% CI 1.02–1.67) was associated with a higher likelihood of on track social-emotional development. Additionally, access to books was associated with a higher likelihood of on track social-emotional development among children in rural areas, but no evidence of an association was found between access to books and social-emotional development among children in urban areas (Fig. 2).

### Physical development

In the adjusted analysis, playing with a child was associated with higher odds of on track physical development than not playing with a child (aOR 1.61; 95% CI 1.01–2.55) (Table 5).

In the subgroup analysis by residence type, playing with a child was associated with higher odds of on track physical development among children in urban areas (aOR 3.37; 95% CI 1.68–6.74) but not among those in rural areas (Table 5). However, among children in rural areas, preschool attendance was associated with a lower likelihood of on track physical development (aOR 0.55; 95% CI 0.33–0.92) than not attending preschool. There was no evidence of an association between access to books at home and physical development for both urban and rural children (Fig. 2).

### Learning-cognitive development

In the adjusted analysis, playing with a child was associated with 1.51 (95% CI 1.14–2.00) higher odds of on track learning-cognitive development in the child than not playing with them (Table 5). In the subgroup analysis by residence type, playing with a child was associated with a higher likelihood of on track learning-cognitive development among children in rural areas (aOR 1.69; 95% CI 1.24–2.31) but not among those in urban areas (Table 5). There was no evidence of an association between access to books at home and learning-cognitive development (Fig. 2).

## Discussion

We used data from two large, nationally representative surveys to examine whether caregiver-child engagements, early stimulation, and learning opportunities impact early childhood development. Most children were developmentally on track in the physical (95.0%) and learning-cognitive (86.4%) domains. Only about one-third (36.7%) of the study population was developmentally on track in the literacy-numeracy domain. Notwithstanding, the on track literacy-numeracy prevalence reported in this study is higher than estimates reported for most African countries and other low- and middle-income countries (LMICs)^34–36^. In a pooled analysis of data on about one million children aged 36–59 months in 35 LMICs, only 30% of children were on track in the literacy-numeracy domain^34^. One possible explanation for the lower prevalence of on track literacy-numeracy development is that most children in LMICs, such as Ghana, receive inadequate literacy and numeracy support at home and lack access to early-life educational opportunities to support development in the domain^37,38^. For example, in 2019, UNICEF estimated that over 400,000 pre-schoolers in Ghana were not enrolled in pre-primary education^37^. Also, children have limited access to high-quality educational resources, such as books and playthings, that can help them develop strong literacy and numeracy skills^37,38^.

It is, therefore, not surprising that children who had access to children's books at home and those who attended preschool were more likely to be developmentally on track in the literacy-numeracy domains in this study. Our findings align with several prior studies^10,34,37,38,40–42^. For example, a study of 36–59-month-olds in 35 LMICs found that the likelihood of being on track in literacy-numeracy was nearly two times higher (OR 1.89) for children who had at least one book at home compared to children who had no children's book at home^34^. In an earlier analysis of Multiple Indicator Cluster surveys in Belize, the Dominican Republic, Guyana, Jamaica, and Suriname, the number of books at home and enrolment in early childhood education programmes were positively associated with child development^10^. Having books in the home encourages children to read and fosters an appreciation for literature. It can also prompt shared activities between adults and children. The fact that access to books was associated with social-emotional development in rural areas but not in urban settings underscores the importance of early exposure to books and reading materials in promoting children's social-emotional well-being in rural communities.

The positive impact among children who attended preschool could be because activities and experiences in early childhood education programmes are often tailored to encourage the development of literacy and numeracy skills such as letter and number recognition^37,38,41,43^. These programmes also provide children with a stimulating and supportive learning environment and teachers who support and guide them to ensure they learn at an appropriate pace and in a way that meets their individual needs^38,43^. Furthermore, children enrolled in early childhood education programmes can access materials and resources such as books, puzzles, and playthings that promote their development^38^. The stronger association between preschool attendance and on track literacy-numeracy development in rural areas compared to urban areas underscores the importance of early education programmes in rural communities. It further suggests that investing in quality preschool education can substantially benefit children's literacy and numeracy skills, particularly in rural areas. Nonetheless, our findings showed that preschool attendance among children in rural settings was associated with a lower likelihood of being on track in physical development compared to not attending preschool. This finding raises questions about the effectiveness of preschool programmes in promoting physical development among rural children in Ghana. Further research is needed to explore the reasons behind this association.

We found that engaging in activities such as storytelling, counting, drawing, and reading with children enhances their literacy-numeracy skills. In line with these findings, several previous studies have linked home literacy practices to improved reading abilities^10,11,16,44^. For example, in Kenya, researchers found that when a father reads to a child, that child is about four times more likely to be able to read and two times more likely to identify words than children whose fathers did not read to them^11^. Similarly, in five Caribbean countries, Roopnarine and Dede Yildirim^10^ found that telling stories to children was linked to improved literacy skills. It has been demonstrated that storytelling encourages children's creativity and problem-solving^15,47^. When a child listens to stories with characters and plots outside of their daily experience, their creativity and ability to learn and comprehend new words improve^13,48^. These early learning opportunities and engagements provide children with a solid foundation in vocabulary, language patterns, and word sound recognition^20^. It also allows them to practice their language skills with their parents and other family members. However, it is interesting to note that telling stories and engaging in counting or drawing activities did not show a significant association with literacy-numeracy development in urban settings. The varying impact could be attributed to variations in stories, frequency of engagements, and interactions during storytelling.

In rural areas, where there are more open spaces and unstructured environments, play was associated with learning-cognitive development. However, this association was not observed among urban children. It is possible that differences in context, such as increased access to electronic media in urban areas, reduced the impact of play on cognitive development in those areas. Furthermore, differences in socio-cultural norms and the types of play resources could explain the differential influence of play.

It is possible that going outside the home had a negative association with child social-emotional development because indoor environments in Ghanaian homes offer more opportunities for engagement, stimulation, and social interactions than outdoor settings. In Ghana, households typically include extended family members and their children. The extended family contributes to the upbringing of children, and the task of raising children is shared among grandparents, aunts, and uncles^30,32^. This collective approach to childrearing creates a supportive environment that can enhance the child’s opportunity for engagement and interaction with a diverse group of people, fostering early development. It is also common for multiple families to live in the same compound house^49^ (a house with many rooms, each of which opens to a communal space and is occupied by different families). These arrangements provide more opportunities for children to play and interact with other children of similar ages and adults from different families than taking them outside. This may also help to explain the disparity between the results found in rural areas, where large families often live together and traditional childcare practises are more common, and those found in urban areas, where such practises are less common.

It is important to make caregivers aware of the developmental benefits of these activities and how to implement and incorporate them into daily routines. This could be accomplished through public awareness campaigns and educational initiatives emphasising the advantages of early stimulation and learning opportunities. In addition, the government of Ghana should prioritise investment in quality pre-primary education and ensure preschools and kindergartens have access to age-appropriate resources such as books and toys that promote critical thinking. The government could establish regional and district-level state-subsidised book and toy stores to reduce potential disparities in access to books and toys. Furthermore, pre-schoolers from low-income families who do not have access to books at home would greatly benefit from access to resources at public libraries where they can borrow books to read with their families at home. Increased access to books for children is likely to result in higher achievement of developmental milestones. There is also a need to invest in play parks to provide an environment for children to play and interact with their peers when taken outside. In addition, families who lack space at home for their children to play, listen to stories or engage in other forms of early stimulation could use these parks to provide these opportunities for their children.

One of the major strengths of this study is the use of a large, nationally representative sample generalisable to all Ghanaian children aged 36–59 months. In addition, the data on early childhood development indicators were collected using standardised questionnaires that were field-tested in Ghana and other low-income countries. These questionnaires had high reliability and validity. Also, previous studies on early childhood development may not have adequately captured the unique childrearing practices and experiences unique to Ghana. Early childhood development is strongly influenced by culture, and what is effective in one context may not be in another, highlighting the importance of our study to the development of sustainable and effective interventions tailored to the Ghanaian context. Nevertheless, the study has some limitations. The data on the outcomes and early stimulating activities were based on reports from mothers or primary caregivers. Some respondents may have exaggerated their interactions with their children or the child's abilities. These potential biases could have skewed the estimates we provided. However, any biases will be minimal because the field research assistants were trained to ask questions in a way that minimises recall and desirability biases. Also, the analysis did not control some potential confounders, such as parental attitudes towards education and cultural practices, which could bias our estimates. Due to substantial missing observations (63.7%), we did not also adjust for maternal reports of child size at birth. Future studies should employ prospective designs and investigate the mechanisms and possible moderators underlying the positive associations shown in this study. Additionally, future studies could explore the role of cultural practices, access to healthcare, and nutrition in early childhood development.

## Conclusion

Our study provides important insights into the achievement of four key early childhood developmental milestones. The prevalence of on track development varied across domains, with physical development having the highest prevalence and literacy-numeracy development having the lowest. Our findings highlight the importance of caregiver-child interaction, early childhood education, and access to books in child development. To help children reach their developmental milestones, caregivers should create supportive environments inside and outside the home and provide resources that encourage and facilitate positive interactions and engagements between children and caregivers.

## Data Availability

The data supporting this study's findings are publicly available with UNICEF's MICS programme (https://mics.unicef.org/). Permission and approval were sought from UNICEF's MICS programme to access the data for this study.
